# Leveraging radiomics and AI for precision diagnosis and prognostication of liver malignancies

**DOI:** 10.3389/fonc.2024.1362737

**Published:** 2024-05-08

**Authors:** Maryam Haghshomar, Darren Rodrigues, Aparna Kalyan, Yury Velichko, Amir Borhani

**Affiliations:** Feinberg School of Medicine, Northwestern University, Chicago, IL, United States

**Keywords:** radiomics, AI, liver tumors, HCC, review

## Abstract

Liver tumors, whether primary or metastatic, have emerged as a growing concern with substantial global health implications. Timely identification and characterization of liver tumors are pivotal factors in order to provide optimum treatment. Imaging is a crucial part of the detection of liver tumors; however, conventional imaging has shortcomings in the proper characterization of these tumors which leads to the need for tissue biopsy. Artificial intelligence (AI) and radiomics have recently emerged as investigational opportunities with the potential to enhance the detection and characterization of liver lesions. These advancements offer opportunities for better diagnostic accuracy, prognostication, and thereby improving patient care. In particular, these techniques have the potential to predict the histopathology, genotype, and immunophenotype of tumors based on imaging data, hence providing guidance for personalized treatment of such tumors. In this review, we outline the progression and potential of AI in the field of liver oncology imaging, specifically emphasizing manual radiomic techniques and deep learning-based representations. We discuss how these tools can aid in clinical decision-making challenges. These challenges encompass a broad range of tasks, from prognosticating patient outcomes, differentiating benign treatment-related factors and actual disease progression, recognizing uncommon response patterns, and even predicting the genetic and molecular characteristics of the tumors. Lastly, we discuss the pitfalls, technical limitations and future direction of these AI-based techniques.

## Introduction

Liver tumors, both primary and metastatic, have become a growing global health concern with significant implications. Treating HCC remains challenging given the heterogeneity and complexity of the disease. Most HCC patients have underlying cirrhosis or chronic inflammation. The microscopic changes, in the setting of chronic inflammation, makes HCC an ideal disease state to consider for targeted therapy. While immunotherapy has changed the first line treatment paradigms, there remains a paucity of treatment options in patients who either progress on immunotherapy or are intolerant of these agents. Historically, treatments for HCC have been based on the Barcelona Clinic liver cancer staging system, with the assessment of tumor burden, liver function, and general health status guiding the selection of the best treatment modality (1). However, in the era of precision medicine, tumor biomarkers and treatment selection challenge the one-size-fits-all concept in HCC.

The low sensitivity and specificity of biomarkers has rendered selection of treatment to be difficult. While Alpha-fetoprotein (AFP) has historically been used for detection of early, potentially curable tumors, it is limited by its sensitivity to make treatment decisions (2). Biomarkers that predict response to systemic therapy are urgently needed. Presently, AFP is the only biomarker to predict response, and only in a subset of patients who receive ramucirumab as a second-line agent. Using cell free DNA’s genomic and epigenetic changes potentially offers a more sensitive and promising biomarker, especially for detecting minimal residual disease (2). Genetic changes detected by means of circulating tumor DNA allows improved understanding of tumor biology and disease heterogeneity.

Imaging plays a vital role in detecting liver tumors but sometimes conventional methods often lack the precision needed for proper characterization, leading to the need for invasive tissue biopsy. Conventional imaging methods provide limited information on the prognostic factors of liver tumors, such as genetic mutations, molecular markers, and potential treatment response. This information gap delays personalized treatment planning and prognostication.

Rapid advancements in imaging and post-processing techniques have revolutionized high-throughput image analysis, enabling a more precise and comprehensive evaluation of liver diseases. Artificial intelligence (AI) and radiomics have emerged as promising methods with the potential to revolutionize liver lesion characterization. AI and radiomics can analyze medical images at a high level of detail, identifying subtle patterns that correlate with specific tumor types, stages, and biological characteristics. These methods are fast, affordable and readily available. AI and radiomics can do simple tasks and handle a huge amount of data with the same accuracy, meaning that missing manual steps, fatigue, or data overload won’t affect the findings.

We provide an outline of radiomics and AI contributions to diagnosis and staging, treatment response assessment, and prognosis prediction in liver malignancies in this review. We describe the progress and potential of AI in the liver oncology imaging, focusing specifically on radiomic and deep-learning techniques.

## Radiomics and artificial intelligence

**Figure 1:**
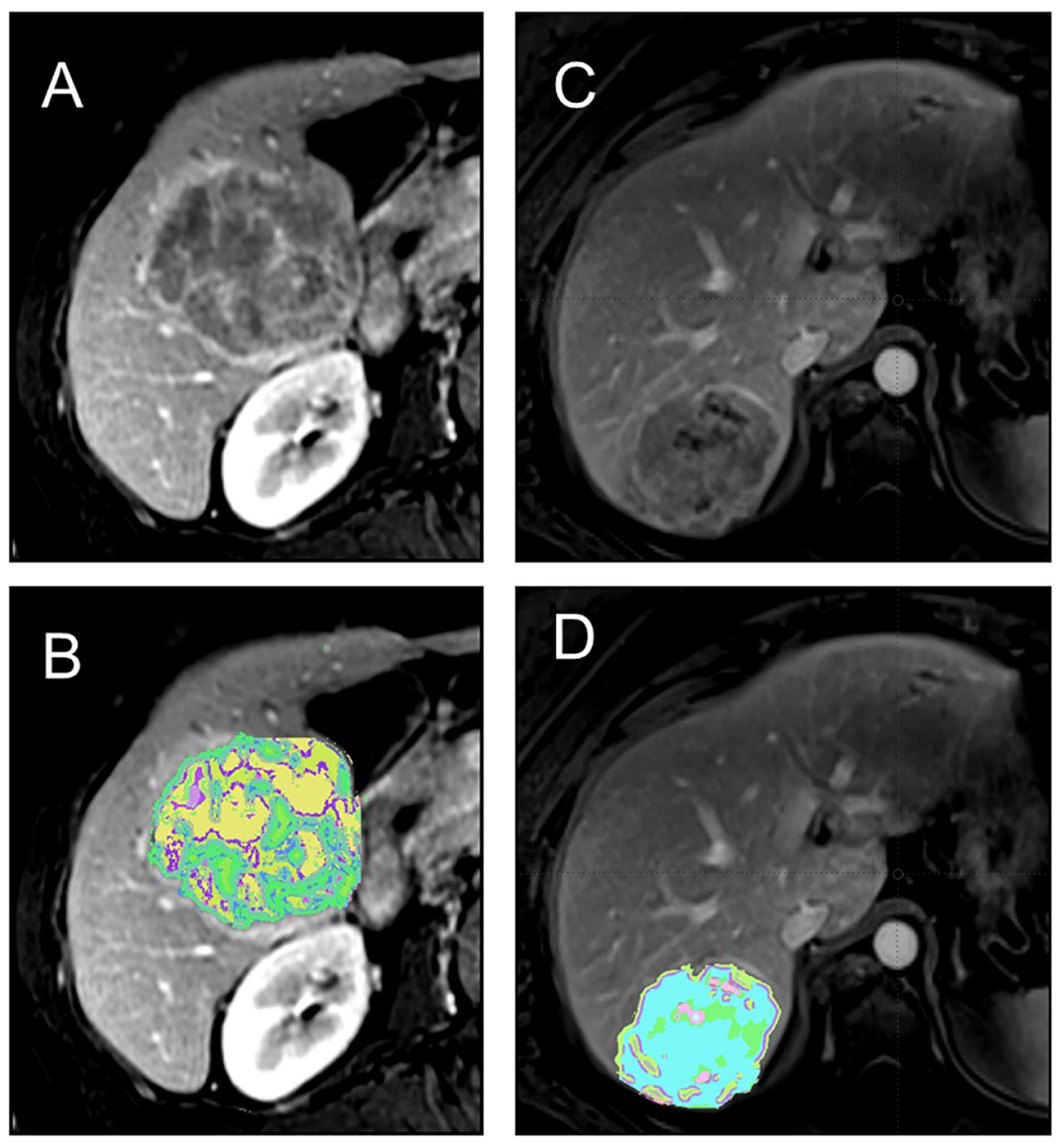
**(A, B)** 64 y/o M with history of cirrhosis and HCC.Contrast-enhanced T1-weighted MRI shows a heterogeneous tumor **(A)** with associated texture heterogeneity map demonstrating tumor habitats **(B)**. The patient had poor outcome with several recurrent lesions after surgical resection suggestive of poor tumor biology. **(C, D)** 54 y/o F with history of cirrhosis and HCC. Contrast-enhanced T1-weighted MRI shows a less heterogeneous tumor **(C)** with associated texture heterogeneity map showing the tumor habitats **(D)**. The patient good outome after resection with no recurrence.

Radiomics, a framework that complements conventional radiological interpretation, has emerged as a powerful tool for extracting and quantifying texture characteristics derived from tumor heterogeneity (3–6) (**Figure 1**). Radiomics employs a wide range of method, each designed to capture specific aspects of tissue architecture and texture. These features provide a detailed representation of tumor heterogeneity, enabling researchers to study and compare tumor characteristics across different patients. The standardization of radiomics features ensures the reproducibility and generalizability of radiomics studies, fostering collaboration and wider adoption of this technique. Development of openly available imaging datasets further creates opportunities to test and benchmark radiomics algorithms and facilitate the translation of radiomics findings into the clinical practice.

Radiomics features extracted from large datasets enable the development of advanced statistical models, including machine learning and artificial intelligence algorithms. These models can enhance various aspects of liver imaging assessment, including tumor origin identification, therapy response prediction, and prognosis assessment. For instance, radiomics provides valuable insights into tumor characteristics, such as aggressiveness and prognosis, which can inform treatment decisions. Another example includes delta-radiomic models, which allow for longitudinal assessment of changes in tumor texture to assess tumor response to treatment. This enables timely adjustments to treatment regimens and improves overall treatment efficacy. Furthermore, radiomics-based predictive models can personalize treatment strategies for individual patients, tailoring treatment to their specific tumor characteristics and maximizing treatment success.

Harnessing the power of neural networks, AI in medical imaging extracts intricate patterns from large datasets and can improve informed predictions. The convolution operation, a cornerstone of many neural networks, employs diverse kernels to transform raw data into meaningful representations, enabling neural networks to learn from and make predictions on complex datasets. Deep learning, a powerful subfield of AI, utilizes many interconnected layers that transform information, enabling more sophisticated information processing. Deep learning’s ability to automatically learn features and representations from data stands out as a key strength, eliminating the need for explicit feature engineering by human experts. This capability makes deep learning particularly well-suited for various clinical tasks. For instance, deep learning algorithms can accurately detect and localize objects within images, enabling the identification of anatomical structures or abnormalities in medical scans. Other models can be trained to precisely segment objects in images, allowing for the delineation of organs and lesions. Segmented organs or lesions can be effectively classified into distinct categories, aiding in disease diagnosis and treatment monitoring. Radiomics can be used to identify the origin of segmented lesions. The integration of radiomics with deep learning has emerged as a promising strategy for enhancing classification performance in medical imaging. Deep learning algorithms possess the ability to complement radiomic features with kernel-based features and then extract patterns from the high-dimensional imaging data. This synergistic combination has yielded noticeable advancements in classification accuracy for a wide range of medical imaging tasks.

**Figure 2:**
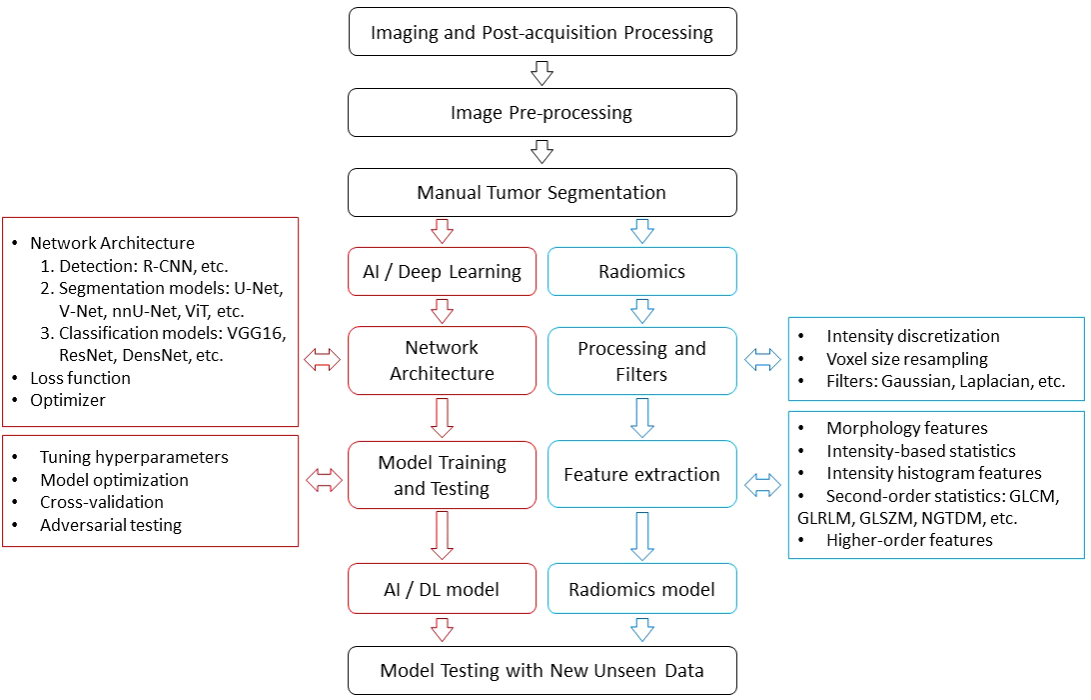
The work flow of radiomics and AI.

While AI-based approaches offer a diverse toolbox for image analysis, both radiomics and deep learning share a similar workflow including collection and standardization of imaging data, image pre-processing, and segmentation of relevant regions depending on the task. In liver disease analysis, this involves whole liver segmentation, segmentation of various structures like lesions, gallbladder, bile ducts, and vascular components. The whole liver segmentation allows for evaluation of liver morphology, liver surface, and parenchymal changes such as fibrosis and cirrhosis. Segmentation and detailed analysis of individual lesions, including their count, size, heterogeneity, necrosis, and vascular involvement, can provide valuable insights for staging, treatment planning, and prognosis (**Figure 2**).

## Segmentation

Segmentation of the liver or its vasculature through CT and MRI has importance in diagnosing lesions, planning radiotherapy, conducting liver vascular surgeries, preparing for liver transplantation, and analyzing tumor vascularization, among other applications. The process of manual segmentation is both time-consuming and susceptible to human errors. Several researchers have explored the use of deep learning models to automate this process, aiming to overcome these limitations. Segmentation studies exhibit remarkable specificity in liver imaging, compared to other fields. The mainstream segmentation technology at present is U-Net, a convolutional neural network (CNN), and its derivatives. The segmentation accuracy for the entire liver, as assessed on the SLIVER07 dataset, is exceptionally high, achieving a maximum Dice Similarity Coefficient (DSC; which is a metric of accuracy of the method) of 0.9827 (7).

Said et al. employed CNNs for HCC segmentation in MRI, achieving fair to good performance, notably excelling in single-slice segmentation with mean DSC ranging from 0.442 to 0.778 in 292 patients (8). Another study focused on automating colorectal liver metastasis and liver ablation zone segmentation on CT, with the Hybrid-WNet model demonstrating high accuracy. Trained on 92 patients, the model achieved a median DSC of 0.73 (9). Additionally, a feasibility study utilized a deep convolutional neural network (DCNN) to automate the application of LI-RADS (Liver Imaging Reporting and data System) algorithm on multiphasic MRI, showcasing efficient liver and HCC segmentation. The average DSC for automatically identified lesions using the DCNN+ random forest classifier employing radiomic features and thresholding was 0.64 in the validation set and 0.68 in the test set (10). One paper presented an automatic algorithm for rapid and accurate liver and lesion segmentation in CT scans. Achieving a 94.2% volume overlap and 3.7 mm accuracy for liver surface segmentation, the method demonstrated a short processing time of 11.4 seconds per slice. Tumor lesion detection showed 82.6% sensitivity and 87.5% specificity (11). A separate study used a deep-learning model for HCC segmentation and classification using gadoxetic acid-enhanced MRI. The 3D U-Net-based model achieved high performance (average DSC of 0.884) for HCC segmentation (12). Lastly, a dual-energy CT radiomics pilot study successfully differentiated between benign and malignant hepatic lesions, outperforming iodine quantification. This involved semiautomatic segmentation of both the volume and rim of individual liver lesions, along with extracting contrast enhancement, iodine concentrations, and radiomic features from each image (13).

Deep learning models can perform HCC segmentation with a high accuracy. This has multiple clinical applications. These models can handle a huge amount of data with the same accuracy, meaning that missing manual steps, fatigue, or data overload won’t affect the findings. Computer-based processing is affordable and readily availability.

## Early detection and accurate tumor classification

Identifying liver cancers in their early stages, when they are localized and amenable to curative treatment, is the ultimate goal. Ideally, the cancer should be diagnosed when patient is asymptomatic as the emergence of symptoms often signifies late-stage, incurable disease in many cases. Moreover, early small tumors should be characterized correctly to allow for correct treatment since different tumor pathologies have distinct course and different treatment approaches. Unfortunately, primary liver cancers may have nonspecific imaging features in their early stages due to their smaller size. Equally significant is the early identification of liver metastases, highlighting the importance timely and precise detection. At the same time addressing and preventing false positives, overdetection, overdiagnosis, and overtreatment is essential (14). As an example, combined HCC and cholangiocarcinoma (CC) originates from hepatic progenitor cells and can display both hepatocytic and cholangiocytic differentiation (15, 16). HCC the most common hepatic malignancy is histologically derived from the hepatocytes and CC the second most common hepatic malignancy is derived from the biliary epithelial cells. Studies have demonstrated that the clinical features and prognosis of combined HCC-CC markedly differ from those of intrahepatic CC (IHCC) and HCC (17, 18). Hence, it holds significant clinical implications to differentiate these tumors preoperatively accurately.

Radiomics integrated with machine learning algorithms has promising role in distinguishing diverse focal hepatic lesions. The features extracted may allow for noninvasive diagnosis and characterization of liver malignancies and provide vital details such as microvascular invasion within tumors. AI has also been extensively employed for classifying different liver lesions. CNNs specifically designed for image recognition tasks have attracted considerable attention for liver cancer diagnosis.

Numerous large scale studies utilizing CT or MR imaging have employed radiomics to distinguish various liver lesions, yielding areas under ROC curves (AUC) ranging from 0.7 to 0.95 (19–29). These investigations demonstrated robust performance not only on the training set but also on testing and validation sets. The scope of these studies encompassed a wide range of classification tasks and discriminating lesions, including HCC, hemangioma, cysts, adenoma, hepatic focal nodular hyperplasia, CC, combined HCC-CC, inflammatory masses, and metastasis. Clinical variables were integrated into certain models to enhance their performance (19–29). A multitude of AI studies has endeavored to predict liver malignancies, focusing on diverse aspects such as detecting HCC (30–32), classifying major features of LI-RADS (12, 33, 34), and discerning classic HCC form other malignant and nonmalignant liver lesions. AUC values in either the training or validation sets varied from 0.6 to 0.942 (12, 30–34).

Presence of microvascular invasion (MVI) is identified as an independent risk factor for the postoperative recurrence of HCC (35). The definitive assessment for MVI is based on histologic examination of surgical specimen, which is only available after resection of tumor. As a result, assessing the MVI status before surgery will play a crucial role in guiding decisions regarding the optimal extent of surgical resection or ablation treatment for individuals with HCC. Several studies using AI or radiomic features extracted from gadoxetic acid-enhanced MRI, dynamic contrast enhanced MR, or contrast enhanced CT images tried to predict microvascular invasion in HCC and mass-forming CC (36). The AUCs ranged from 0.75 to 0.98 with most of the studies achieving AUCs higher than 0.85 (36–47). Notably, studies focused on peritumoral areas within the 5 cm to 10 cm range. One study underscored that patients without MVI experienced significantly prolonged recurrence-free survival (RFS). Validation sets were incorporated in all studies (36–47). As mentioned above, accurately predicting MVI before surgery can significantly influence surgical planning, including decisions regarding the extent of resection or the suitability of ablation treatments. Such high AUCs and predictive capabilities mean that presence of MVI can be successfully determined with AI and radiomics prior to surgery allowing for a more personalized surgical approach, potentially improving postoperative outcomes and recurrence-free survival for patients with HCC.

## Grading, association with molecular profile, immunophenotype, etc.

HCC histopathological grading has been identified to be closely associated with the prognosis of the tumor, serving as an indicator of the tumor’s biological behavior. Extensive research indicates that both progression-free survival and overall survival are notably lower in poorly differentiated HCC compared to well-differentiated HCC. Certain subtypes of HCCs, such as macrotrabecular-massive subtype, are also correlated with worse prognosis. Pre-operative knowledge of tumor grading affects treatment plan and surgical approach, when surgery is indicated. For example, the recommendation is to opt for an extended resection margin when conducting liver surgery for poorly differentiated HCC to minimized the risk of early recurrence. Some data suggest recommendation against liver transplantation for patients with HCC that is both poorly differentiated and exceeds 3 cm in size. Preoperative knowledge of tumor grading is classically achieved by histologic examination of biopsy specimen. Biopsy however is an invasive procedure and is not feasible in all patients (due to patient’s factors and location/size of the tumor). Additionally, given the high success of imaging-based criteria for noninvasive diagnosis of HCC (such as LI-RADS criteria), biopsy is not routinely performed in this population.

Several radiomics models utilizing gadoxetic acid-enhanced MRI, some augmented by AI, have aimed for HCC subtyping and grading to overcome these issues. They have achieved AUCs ranging from 0.6 to 0.912 (48–53). Notably, lower grades were correlated with longer progression-free survival in one cohort. Additionally, the radiomics model demonstrated associations with dysregulated humoral immunity, encompassing B-cell infiltration and immunoglobulin synthesis, offering valuable insights into the immune microenvironment of HCC (48–53).

Comprehensive knowledge of the molecular profile and immunophenotype of liver cancers is also relevant for advancing precision oncology. The tumor microenvironment and immune status are integral factors influencing the success of immunotherapies and locoregional treatments in HCC (54). Gene expression analysis has revealed distinct immune classes among HCC patients and immune profiling of HCC can predict response to immunotherapy (55). Preliminary works have indicated the potential of radiomics quantification in immune profiling for HCC. Notably, these works studied expression of vascular endothelial growth factor (VEGF) (56), angiopoietin-2 (57), Forkhead Box M1 (FOXM1) (58), and Ki-67 (59, 60). Additionally, the presence of β-catenin mutation (61), intra-tumoral tertiary lymphoid structures (62), cytokeratin 19 (63, 64), glypican-3 (GPC3) (65), immunohistochemical cell type markers for T-cells (CD3), macrophages (CD68) and endothelial cells (CD31), PD1 and CTLA4 at mRNA expression level (66), as well as density of CD3+ and CD8+ T cells (67) were studied. All the aforementioned molecules have relevant task in carcinogenesis. VEGF and Angiopoietin-2 regulate tumor growth by influencing angiogenesis. FOXM1 governs cell cycle genes, Ki-67 marks proliferation, and β-Catenin mutation leads to uncontrolled cell growth. Intra-tumoral Tertiary Lymphoid Structures impact the anti-tumor immune response. Cytokeratin 19 maintains cell structure, while GPC3 serves as a tumor diagnostic marker. Immune cell markers like CD3, CD68, and CD31 reveal cell distribution and density, reflecting the local immune response. PD1 and CTLA4 mRNA levels influence responses to checkpoint inhibitors. Each of these immune subtypes plays a critical role in unraveling the complex immune response within HCC, providing insights for prognostication and targeted therapeutic interventions. AUCs of these tasks fell somewhere between 0.76 to 0.95 (56–67). Notably, when clinical factors were integrated with radiomics signatures, models’ performance significantly improved. In the MRI studies, the hepatobiliary phase consistently demonstrated the best performance.

While deep learning models haven’t been as widely applied as radiomics for this particular task, they undoubtedly hold significant potential. Xie et al. introduced a non-invasive method for predicting PD-1 and PD-L1 expression in HCC. Using a cohort of 87 HCC patients and analyzing 3094 CT images, the Contrastive Learning Network (CLNet) was proposed. Trained with self-supervised contrastive learning, CLNet achieved superior performance, demonstrated an AUC of 86.6 for PD-1 expression and 83.9 for PD-L1 expression (68) (**Table 1**).

**Table 1:** Summary of HCC grading, molecular profiling, and immunophenotyping.

Author	Marker	Subjects	Modality	Model	Accuracy training	Accuracy testing	Accuracy validation
Chen	Immunoscore	207 HCC	MRI	Radiomics/ML	AUC, accuracy, sensitivity, specificity = 0.904, 0.787, 93.8%, 74.6% combined- AUC = 0.926	NA	AUC, accuracy, sensitivity, specificity = 0.899, 0.772, 92.3%, 72.7% combined- AUC = 0.934
Chen	FOXM1 expression	286 HCC	CT	Radiomics/ML	AUC = 0.918	AUC = 0.837	NA
Fan	VEGF expression	202 HCC	MRI	Radiomics/ML	AUC = 0.892 combined- AUC = 0.936	AUC: 0.8/combined- AUC = 0.836	NA
Hectors	Immunoprofiling and genomics	48 HCC	MRI	Radiomics/ML	Tumor size ≥ 5 cm - HCC recurrence (OR = 3.01, p = 0.004, AUC = 0.76).	NA	NA
Li	Intra-tumoral tertiary lymphoid structures	142 HCC	CT	Radiomics/ML	AUC = 0.79	NA	AUC = 0.75
Wang	cytokeratin 19 expression	227 HCC	MRI	Radiomics/ML	AUC = 0.892 combined- AUC, sensitivity, specificity, C-index = 0.951, 0.818, 0.974, 0.959	NA	AUC = 0.73 combined- AUC, sensitivity, specificity, C-index = 0.822, 0.769, 0.818, 0.846
Wu	Ki-67 expression	172 HCC	CT	Radiomics/ML	AUC = 0.854 combined- AUC = 0.884	NA	AUC: 0.744 combined- AUC = 0.819
Yan	Ki67 expression	110 HCC	MRI	Radiomics/ML	AUC = 0.833 combined- AUC = 0.901	NA	AUC: 0.772 combined- AUC = 0.781
Zeng	β-catenin mutation	98 HCC	MRI	Radiomics/ML	AUC, accuracy, sensitivity, specificity = 0.86, 0.75, 1.0, 0.65 combined- AUC = 0.86	NA	AUC, accuracy, sensitivity, specificity = 0.82, 0.73, 0.67, 0.76 combined- AUC = 0.76
Zhang	cytokeratin 19 expression	311 HCC	MRI	Radiomics/ML	C-index, 0.914	C-index, 0.855	C-index, 0.795
Zhang	glypican-3 expression	137 HCC	MRI	Radiomics/ML	AUC, sensitivity, specificity = 0.822, 0.816, 0.706 combined- AUC, sensitivity, specificity = 0.888, 0.777, 0.912	NA	combined- AUC, sensitivity, specificity = 0.800, 0.58.5, 1.0
Zheng	angiopoietin-2 expression	52 HCC	MRI	Radiomics/ML	AUC = 0.8 combined- AUC = 0.933	NA	NA
Xie	PD-1 and PD-L1 expression	87 HCC	CT	AI-DL	AUC = 0.866 for PD-1 expression AUC = 0.839 for PD-L1 expression	NA	NA

High AUC values in both radiomics and deep learning tasks indicate strong predictive performance, meaning these models are highly effective in identifying molecular profiles, immunophenotypes and grades of HCC.

## Assessment of tumor response

Several locoregional therapeutic strategies have been developed and implemented over past decades, and a considerable number of these are currently considered as the standard of care for liver malignancies (69). These involve a range of percutaneous and trans-arterial methods designed to induce cell death in tumors. This can be achieved through percutaneous approach, as seen in radiofrequency and microwave ablation, or achieved via targeted trans-catheter trans-arterial administration of embolic agents (known as trans-arterial bland embolization, TAE), chemotherapeutic substances (referred to as trans-arterial chemoembolization, TACE), or radioembolizing agents (as in trans-arterial Yttrium-90 radioembolization, TARE) (70). While typically less invasive compared to surgical removal and transplantation, these therapies can lead to complications. Hence, careful patient selection and thorough evaluation of treatment response are crucial clinical considerations. Evaluating the response to treatment following TARE and external beam radiation, particularly in the initial months post-treatment, poses challenges due to the intrinsic characteristics and timeline of cytotoxic effects induced by radiation. Sustained enhancement in the arterial phase and subsequent washout can be observed in treated lesions for several months following the treatment, even though a complete response is ultimately achieved (71). Considering these limitations, it is essential to explore alternative approaches for evaluating treatment response. Numerous studies have explored the potential of utilizing radiomic features extracted from post-treatment CT and MRI in assessing the treatment response of HCC (72–79). There are limited studies on other liver tumors (80, 81). Radiomics features were extracted from diverse imaging modalities, including MRI and CT scans, and involved different treatment methods. In some studies key clinical information, such as albumin levels, AFP levels, and Child-Pugh score were integrated into predictive models to enhance accuracy. The studies anticipated diverse outcomes encompassing early response, early recurrence, aggressive intrasegmental recurrence, tumor refractoriness, and local tumor progression across varied locoregional strategies. The AUC values of these studies ranged from 0.8 to 0.95 (72–81). These studies collectively underscore the potential of radiomics in tailoring treatment strategies and prognostic assessments for liver cancer patients, providing a non-invasive means to predict outcomes and guide personalized interventions based on comprehensive imaging analyses and relevant clinical parameters.

The utilization of deep learning to evaluate locoregional therapeutic responses in HCC is relatively limited in the current body of research, yet, it’s important to note that the studies presented are novel, and there’s considerable unexploited potential in this evolving field. Three studies employed deep learning to explore the response of TACE in HCC (82–84). In a study involving 414 patients, hazard ratios for time to progression (TTP) were 0.32 (training), 0.28 (validation), and 0.55 (test). The research also indicated improved overall survival (OS) with a hazard ratio of 0.58 and a median survival of 38.8 months, compared to 20.9 months (82). Another investigation with 789 patients achieved an 84.3% accuracy, showing AUCs of 0.97, 0.96, 0.95, and 0.96 for complete response (CR), partial response (PR), stable disease (SD), and progressive disease (PD), respectively. The deep learning model displayed accuracies of 85.1% and 82.8% across CR, PR, SD, and PD in two validation sets (83). The deep learning signature showed strong predictive performance, with a C-index of 0.717 in the training set and 0.714 in the validation set (84).

One study developed an automatic and non-invasive deep learning radiomic nomogram (DLRN) to predict hepatic arterial infusion chemotherapy response in HCC. Utilizing contrast-enhanced CT images from 458 patients across three hospitals, the DLRN achieved high AUC values of 0.988 (training), 0.915 (internal validation), and 0.896 (external validation), outperforming other models. The DLRN also successfully stratified survival risk, with the predictive objective response group exhibiting significantly longer overall survival (26.0 vs. 12.3 months) (85).

The ability of AI and radiomics to predict early treatment response and recurrence can improve the management of liver cancer. By identifying patients at risk of aggressive recurrence or poor response to treatment early on, clinicians can adjust therapeutic strategies promptly have shown that these methods have consistently high accuracies.

## Prognostication

The prognosis of HCC continues to be unfavorable, even following curative-intent treatments like liver resection or transplantation. After liver resection, the early recurrence rate stands at 50–70%, while following a median post-transplant period of 13–14 months, the rate is 10–20% (86, 87).

By analyzing detailed features from medical images, radiomics helps predict recurrence, understand tumor growth, and estimate progression-free survival. When combined with essential clinical details, radiomics can become a powerful tool for predicting aggressive disease and customizing treatments. This approach offers a non-invasive, precise way to enhance prognostic assessments, bringing a new level of accuracy to liver cancer care.

Multiple studies - including ten studies on HCC (88–97), four studies on Mass-forming CC (98–101), and three studies on colorectal liver metastases (102–104)- utilized various radiomics approaches to predict outcomes and guide treatment decisions. The studies involved diverse cohorts, including patients undergoing liver transplantation, surgical resection, or chemotherapy. The endpoint outcomes ranged from overall survival (OS), recurrence free survival (RFS), progression-free survival (PFS), event-free survival (EFS), early recurrence (ER), 1-year survival and 5-year survival, post-hepatectomy liver failure (PHLF), and lymph node metastasis. The AUCs for predictive models varied, ranging between 0.70 to 0.98 (88–104). Moreover, the integration of radiomics with clinical factors consistently improved predictive performance, demonstrating the potential for personalized risk assessment. Notably, radiomics models were applied to predict survival in various contexts, offering valuable insights for prognosis and treatment planning in HCC and other liver cancers.

Radiomics analysis and the integration of CNNs with CT and MRI images and clinical data have been developed to predict the prognosis of HCC patients. Machine learning and CNNs have exhibited a good accuracy in predicting patient survival following surgical treatment. In a bicentric study, a deep learning nomogram based on gadoxetic acid MRI features was developed to predict early recurrence in 285 HCC patients post-hepatectomy. Extracting deep learning features using VGGNet-19 from contrast-enhanced MRI images, the deep learning nomogram, incorporating multiphase deep learning signatures, performed well on both the training (AUC: 0.949) and validation sets (AUC: 0.909). Independent predictors for early recurrence included microvascular invasion, tumor number, and the deep learning signature (105). Lv et al. introduced an AI -powered approach for predicting the 3-year recurrence of HCC using contrast-enhanced CT radiomic profiles. In a single-center retrospective cohort of 224 HCC patients, radiomic signatures from arterial and portal venous phases were utilized to establish three models: radiological model (RM), deep learning-based radiomics model (DLRM), and clinical & deep learning-based radiomics model (CDLRM). CDLRM, incorporating clinical factors and DLR features, demonstrated superior accuracy (AUC: 0.98 in training, 0.83 in testing) compared to DLRM and RM (106). In a proof-of-concept study for HCC patients initially eligible for liver transplant, machine learning models were developed using pretreatment clinical and MRI features to predict posttreatment recurrence. The study included 120 patients, and three machine learning models (clinical, imaging, combined) predicted recurrence with AUCs ranging from 0.60 to 0.86 across six timeframes. The imaging model outperformed the clinical model (mean AUC 0.76 vs. 0.68, p = 0.03). Kaplan-Meier analysis demonstrated significant differences in recurrence risk prediction between low and high-risk groups for all three models (107). A retrospective study, involving 55 patients with stage 4 colon cancer and hepatic metastasis, explored the role of MRI-based measures of intra-tumor heterogeneity in predicting survival. Extracting a heterogeneity phenotype vector from 94 hepatic lesions, the study identified 22 texture features associated with patient survival. A random forest machine learning model, combining clinical variables with imaging-based features, improved survival prediction performance, yielding an area under the ROC curve of 0.94 compared to 0.83 with clinical variables alone (108).

By analyzing the complex patterns within imaging data, these approaches allow for a deeper understanding of tumor biology and patient-specific disease progression. The predictive capability of radiomics and AI models, as evidenced by their high accuracy in various studies, emphasizes the need for ongoing research to further validate and integrate these technologies into clinical practice.

## Pitfalls and technical limitations

Despite the promising results in radiomics research for liver cancers, a notable gap persists between numerous numerical data generated and their practical clinical application. These studies provide a myriad of quantitative metrics and predictive models, showcasing radiomics’ potential in augmenting diagnostic and prognostic evaluations. However, translation of these findings into routine clinical practice remains uncertain. Challenges, including protocol variability and interobserver discrepancies, present significant obstacles in bridging the research-clinical gap. Noteworthy is the absence of clear guidelines on the integration of radiomic data into the real-world clinical decision-making. The intrinsic heterogeneity of liver tumors and the dynamic nature of cancer progression amplify the intricacies of developing robust and generalizable radiomic models. Additionally, challenges related to overfitting, model validation, and potential false correlations in high-dimensional data emphasize the need for rigorous methodology standardization. These technical challenges collectively underscore the substantial work required before radiomics can claim its role as a dependable and clinically impactful tool in liver cancer management.

While machine learning has shown remarkable promise in the radiologic assessment of primary and metastatic liver malignancies, it is not without its pitfalls. One significant challenge lies in the quality and quantity of training data. The performance of machine learning models heavily relies on the availability of diverse and representative datasets, and issues such as imbalances, biases, or insufficient samples can lead to suboptimal generalization and performance. Additionally, the interpretability of machine learning models in radiology remains a concern. The “black-box” nature of some sophisticated algorithms makes it challenging for clinicians to understand the rationale behind specific predictions, limiting their trust and acceptance. Another notable pitfall is the potential for overfitting, where a model may perform exceptionally well on the training data but fails to generalize effectively to new, unseen cases. Moreover, the dynamic nature of medical imaging and evolving standards in radiologic practices pose challenges in keeping machine learning models up-to-date and adaptable to changes in the field. Addressing these pitfalls is crucial to harness the full potential of machine learning in improving the accuracy and efficiency of radiologic assessments for liver malignancies.

## Future direction

It’s important to acknowledge the gap between research advancements in radiomics and AI and their clinical implementation. This gap mainly exists because the low external validity of these technologies limits their adoption in routine clinical practice. The primary challenge for clinical translation is ensuring the generalizability of AI and radiomics models. There is a need for further clarification of true role of radiomics and machine learning tools in clinical applications. This involves external validation of machine learning models and the assessment of diagnostic performance for specific diseases using deep learning radiomics. External validation, particularly through large multi-institutional datasets gathered over a longer period, is essential to confirm the models’ generalizability. To enhance the clinical translation and applicability of radiomics studies, it is also crucial to address important issues such as access, cost-effectiveness analysis, and the promotion of open data practices. Generally, achieving sufficient clinical performance in training a CNN necessitates a large amount of training data. In the development of AI imaging models, the cost of annotation is a significant concern, and the future is expected to see a focus on acquiring substantial amounts of high-quality training data while simultaneously minimizing annotation costs. The ultimate goal is to leverage AI and radiomics in clinic for the precise classification and detection of liver tumors and to enable personalized treatment by accurately predicting treatment responses.

## Conclusion

In this review we identified several potentials of AI and radiomics in clinical decision-making in liver oncology imaging, including improving the precision of tumor detection, characterization and classification, enabling the prediction of treatment response, identifying patient-specific prognostic indicators for personalized therapy, and possibly reducing the reliance on invasive procedures like biopsies by non-invasively determining tumor genetics, immune phenotype and behavior.

## Author contributions

MH: Writing – original draft. DR: Writing – original draft. AK: Writing – original draft. YV: Writing – original draft, Writing – review & editing. AB: Writing – original draft, Writing – review & editing.
